# Supplementary figures and images for: Uncovering the genomic and metagenomic research potential in old ethanol-preserved snakes
Source: PLoS One. 2021 Aug 23;16(8):e0256353. doi: 10.1371/journal.pone.0256353 (PMC8382189; doi:10.1371/journal.pone.0256353)

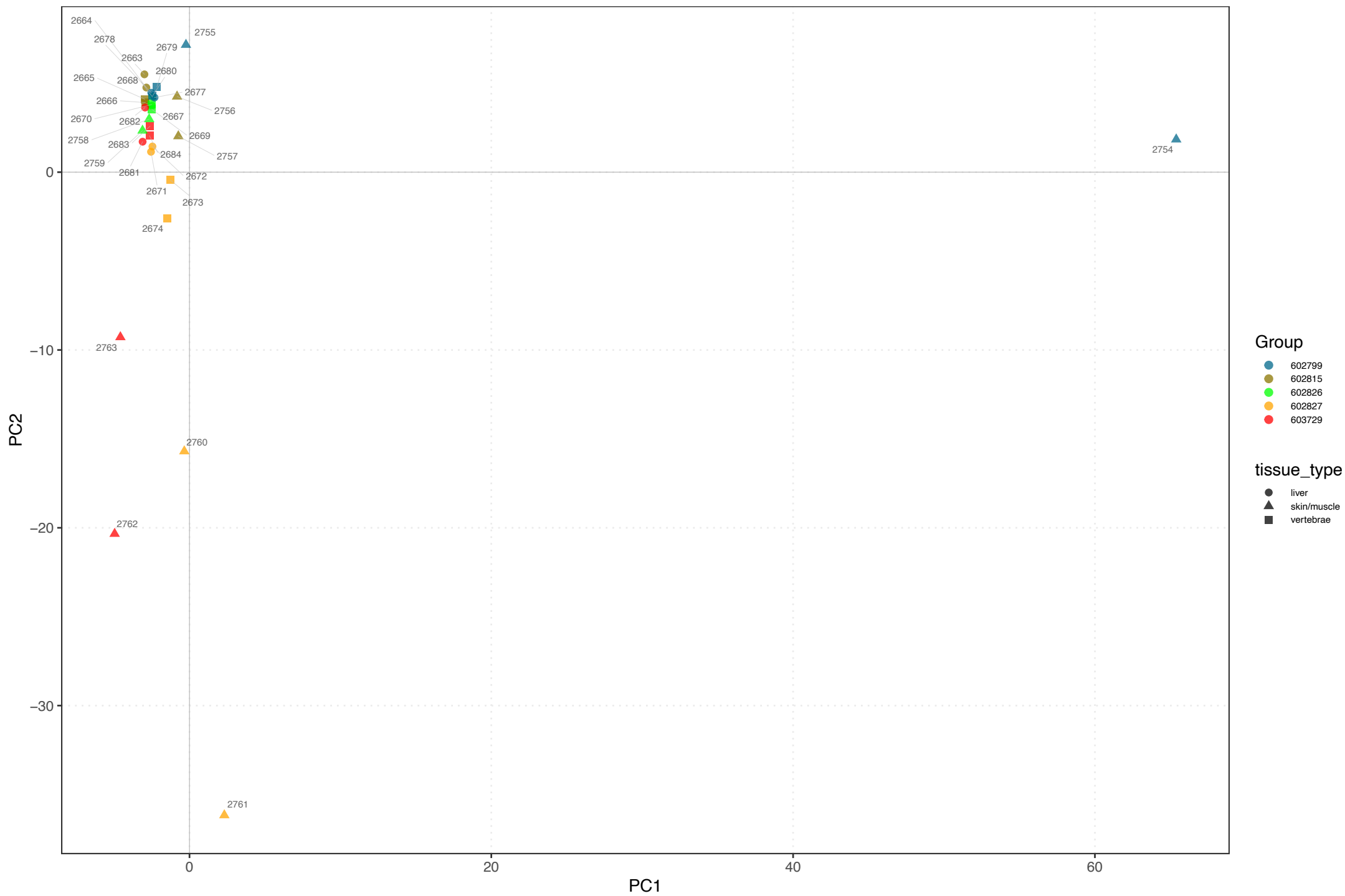

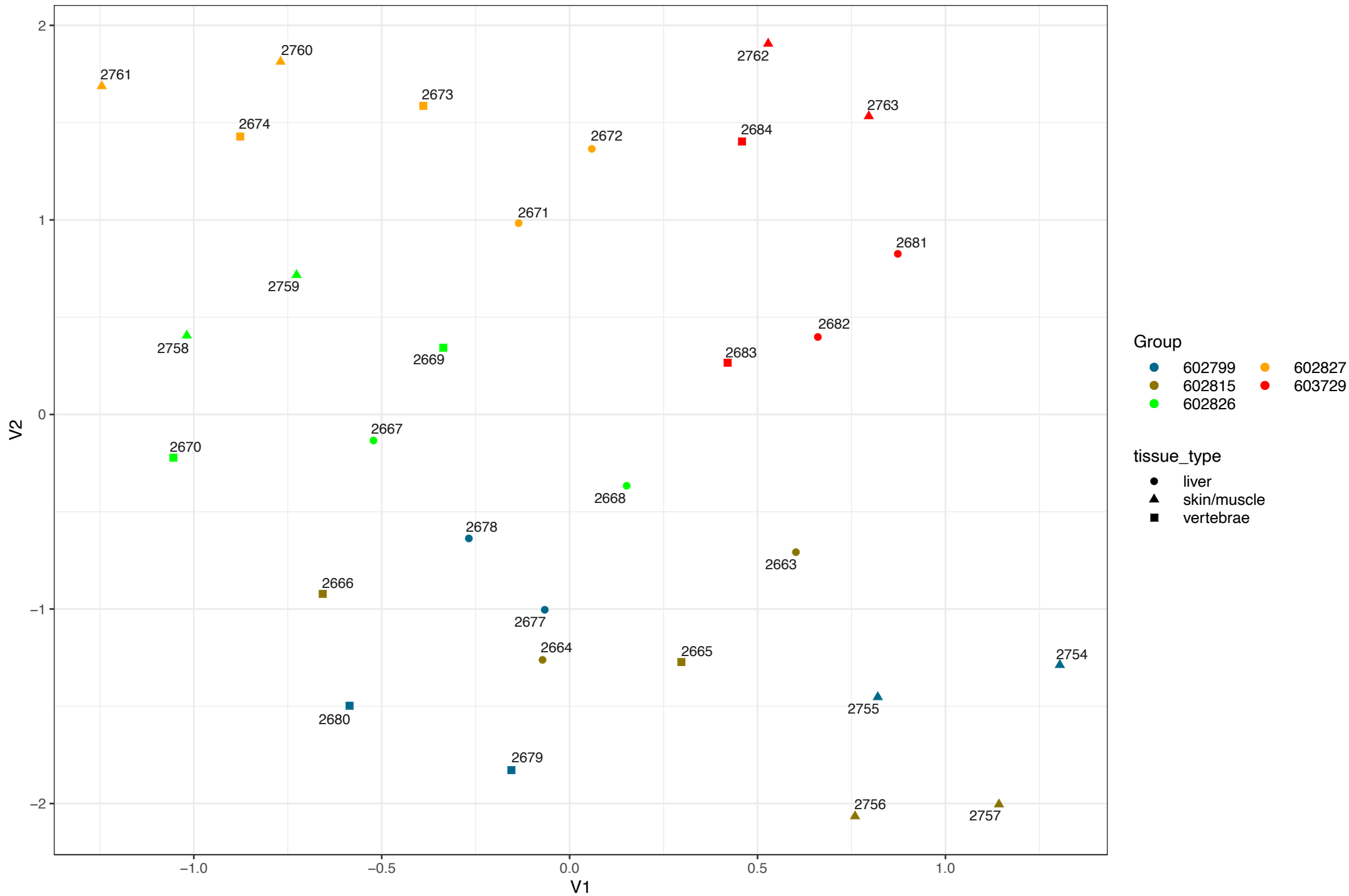

Supplement: S1 Fig — PCA analysis based on weighted number of hits obtained from the Kraken2/Bracken classification for each extract. The weighting was done by transforming the raw number of classified hits into fractions to account for differences between total number of DNA reads among the samples. The different types of tissue are noted. Uniform Manifold Approximation and Projection (UMAP), was used to add further dimensionality in order to assess the relationship between the metagenomic profiles from all tissues and individuals. Blanks were excluded for the analysis. (PDF) [file pone.0256353.s001.pdf]
