# Supplementary material for: Uncovering the genomic and metagenomic research potential in old ethanol-preserved snakes
Source: PLoS One. 2021 Aug 23;16(8):e0256353. doi: 10.1371/journal.pone.0256353 (PMC8382189; doi:10.1371/journal.pone.0256353)
Supplement: S1 Table — Results of formalin test using two test methods. (DOCX) [file pone.0256353.s002.docx]

| **Specimen** | **Test A** | **Test B** | **Formalin present** |
| --- | --- | --- | --- |
|  | 55 ^o^C , stirred, 0,5 x 0,5 x 0,5 cm in 20 ml 10 % Savinase, after 3 h | 55 ^o^C, no stirring. 0,5 x 0,5 x 0,5 cm in 20 ml 10 % Savinase, after 24 h |  |
| **ZMUC R 602799** | Partly degraded. Fluid unclear, precipitate | Partly degraded, precipitate with larger fragments | Possibly formalin |
| **Partly degraded** |  |  |  |
| **ZMUC R 602815** | Partly degraded – fluid clear, precipitate of small fibrous fragments | Degraded, small fibers | Possibly formalin |
| **Partly degraded** |  |  |  |
| **ZMUC R 602826** | Degraded, almost clear, a little precipitate | Degraded, precipitate with small fibrous fragments | No formalin |
| **Degraded** |  |  |  |
| **ZMUC R 602827** | Not degraded, still larger fragments | Not degraded. Did not fall apart during stirring | Formalin |
| **Not degraded** |  |  |  |
| **ZMUC R 603729** | Partly degraded, fluid unclear, large and small fragments | Partly degraded, fell apart but still larger fragments | Possibly formalin |
| **Partly degraded** |  |  |  |
