# Supplementary material for: Uncovering the genomic and metagenomic research potential in old ethanol-preserved snakes
Source: PLoS One. 2021 Aug 23;16(8):e0256353. doi: 10.1371/journal.pone.0256353 (PMC8382189; doi:10.1371/journal.pone.0256353)
Supplement: Attachment — Submitted filename: Zacho_PLOS_01Apr21.docx [file pone.0256353.s006.docx]

Zacho et al. PLOS ONE

Uncovering the genomic and metagenomic research potential in old ethanol-preserved snakes

This manuscript examines DNA degradation in old museum preserved samples. They sample three different tissue types, skin, bone and liver, from five common garter snake (*Thamnophis sirtalis*) samples. These samples have been collected in different regions and different times, which allows them to examine different preservation techniques and the impacts of sample age on degradation. The authors use ancient DNA protocols and shotgun sequencing to determine the best collection, extraction and sequencing procedures for the analysis of museum specimens. Multiple different metrics, including DNA concentration, percent endogenous DNA, fragment length, C-to-T deamination damage and percent human contamination, are used and the authors find similar results across tissue types. In general, I found this manuscript to be of interest and a valuable contribution to the field. However, I did have several concerns with the manuscript that should be addressed.

Major Comments:

1. The main question of the manuscript was to determine the best approach for sequencing museum specimens and compared multiple different tissues. I believe that these analyses should be more rigorous and should include statistical analyses. The authors compare multiple metrics (Figs. 1 and 2) across tissues and specimens and the addition of statistical comparisons through an ANOVA or a similar analysis would greatly improve the manuscript. This would allow the authors to statistically compare the different tissues within a sample. Further, I believe that statistically testing for differences across samples (ie. comparing liver samples across all specimens) would also strengthen the paper. This would allow the authors to compare the ages of the samples as well as the different storage and preparation techniques used by museums.
2. Further to the comment above, the authors should include standard deviations in the manuscript when they are using averages.
3. The manuscript is relatively well written, but I believe there are 3 areas that would help improve the clarity of the study:
   a. The introduction is quite clear but the last 3 paragraphs need to be more concise. These paragraphs could be combined into 2 paragraphs that would be clearer. The first paragraph should address the similarities to aDNA and the availability of the metagenomic profile. The second paragraph should clearly introduce the objectives and methods of the study.
   b. The discussion is quite long and needs to be shortened for clarity. Each of the sections within the discussion should be clearer and also clearly refer back to previous published work. Also, more information should be provided on the potential benefit of metagenomic analyses with museum specimens. Finally, the conclusion section should be clearer with distinct recommendations for best collection, preservation and sequencing approaches with museum specimens.
   c. The PCA and UMAP analyses are not included in the materials and methods section. Further, one of these ordination plots should be included in the manuscript as they show the differentiation based on sample and tissue type.
4. Higher resolution figures are required. Both Figures 1 and 3 have too low of resolution. The test is not readable in Figure 3.

Minor Comments:

1. The authors should choose a way to refer to the samples and use this consistently throughout the manuscript. The samples are sometimes referred to with the museum ID and sometimes by the year of collection.
2. On L53 the sentence “Despite this, the samples display a relatively high endogenous DNA contents, ranging from 26% to 56%, …” should be changed to “Despite this, the samples displayed a relatively high endogenous DNA content, ranging from 26% to 56%, …”.
3. On L139 the sentence “We used a silica-in-solution extraction method, which have proven highly effective …” should be changed to “We used a silica-in-solution extraction method, which has proven highly effective …”.
4. On L213 the “2000xg” should be changed to “2000×g”. This should also be changed throughout the manuscript.
5. What does the acronym LAF stand for on L217?
6. The measurement “ml” and “μl” should be changed to “mL” and “μL” throughout the manuscript.
7. On L228 the sentence should be changed from “The end-repair step was performed in 25,5 μL reactions using …” to “The end-repair step was performed in 25.5 μL reactions using …”. This change should be made throughout the manuscript.
8. On L238 the sentence “1 μL of library was qPCR quantified with Green …” should be changed to “One microliter of library was qPCR quantified with Green …” as it is the start of a sentence.
9. On L289 the authors refer to a “Supplement Table S2”. This should be changed to “Supplementary Table S2”. This change should be made throughout the manuscript.
10. Further, the names of Table S1 and S2 should be switched as the original “Table S2” is mentioned first in the manuscript.
11. The sentence starting on L365 “These snake specimens are likely to have been handled many times over the years so as proxy for DNA contamination in the various tissues, we mapped the sequencing data against the human reference genome” should also be included in the materials and methods. This is good justification as to why the human reference was used to determine the level of contamination.
12. On L382 the sentence “We focus on the overall differences in exploring …” should be changed to past tense.
13. What do the different colours represent in figure 3?
14. The section in the results starting on L401 looking at the ordination plots needs to be clearer. I agree that in the PCA especially there is differentiation based on tissue type but I am not sure it can be attributed to diversity from these analyses alone.
15. On L436 the sentence “Skin, including snake scales, are partly made of dead keratinized cells [76], why it is not unexpected that they contain lower amounts of DNA than liver tissue” is a fragment.
16. The sentence starting on L457 “Although this is still markedly lower compared to working with fresh tissue, where the endogenous DNA fraction is expected to be close to 100%, we argue that all tissues from the included specimens have adequate levels of endogenous DNA for genome-wide sequencing to be feasible” needs data or references to back up this statement.
17. On L475 “Rat” should not be capitalized.
18. In-text citations should be more consistent. For example, on L476 the sentence reads “Likewise, Allentoft, Rasmussen (13) used an aDNA …” and should be changed to reflect the requirements of PLOS ONE. This should be changed throughout the manuscript.
19. The sentence on L514 “By here mapping the sequences to a reference genome, we can measure the actual endogenous DNA content, and thus document the genomic research potential in this old formalin preserved snake from 1923” is a fragment.
20. The sentence starting on L522 “It is well known that formalin fixation may hamper or completely prevent the successful recovery of DNA from a given sample, but out results show that if indeed the DNA extraction is successful” should be changed to “It is well known that formalin fixation may hamper or completely prevent the successful recovery of DNA from a given sample, but our results show that if indeed the DNA extraction is successful”.
21. The sentence starting on L593 “Using human mitochondrial genomes as reference this was estimated to c. 1% in [80] and 2,3% in [36] and when based on genome-wide shot-gun data, values of 4.3-8.9% [94], <0,3% [35], <0,1% [34], and 0.27 % [33] have been reported” is unclear and needs to be rewritten.
22. References are needed in the paragraph starting on L603 comparing the levels of microbial DNA found in this study to other studies.
23. The sentence starting on L624 “Our metagenomic profiling is intended as a proof-of-concept to evaluate the potential and we caution that the exact taxonomic identification of specific microbial taxa require rigor follow-up investigations” should be changed to “Our metagenomic profiling is intended as a proof-of-concept to evaluate the potential and we caution that the exact taxonomic identification of specific microbial taxa require rigorous follow-up investigations”.
24. The sentence starting on L647 “This could serve as window into past microbial communities related to …” should be changed to “This could serve as a window into past microbial communities related to …”.
